# Nd: YAP Laser in the Elimination of Endodontic Nickel-Titanium Files Fractured in Rooted Canals (Part 1: Teeth With Minimal Root Curvature)

**DOI:** 10.7759/cureus.75276

**Published:** 2024-12-07

**Authors:** Amaury Namour, Marwan El Mobadder, Patrick Matamba, Lucia Misoaga, Delphine Magnin, Praveen Arany, Samir Nammour

**Affiliations:** 1 Department of Dental Sciences, Faculty of Medicine, University of Liege, Liege, BEL; 2 Laser Laboratory, Oral Surgery Department, Wroclaw Medical University, Liege, BEL; 3 Bio- and Soft Matter Division, Institute of Condensed and Nanosciences, Université Catholique de Louvain (UCL), Louvain-la-Neuve, BEL; 4 Department of Oral Biology, Biomedical Engineering and Surgery, University at Buffalo, New York, USA

**Keywords:** endodontics, endodontic treatment, fractured instruments, lasers, nd: yap laser, nickel titanium files, root canal, root canal therapy

## Abstract

Background

Fracture of nickel-titanium (Ni-Ti) instruments in root canals is commonly associated with compromised outcomes in endodontic treatment. There is no single, universally accepted approach for managing this complication. The objective of this study is to evaluate the effectiveness of an Nd: YAP laser-assisted protocol in removing fractured Ni-Ti files in teeth with minimal root curvature (less than 15 degrees).

Methods

A total of 66 notched Ni-Ti files located at the apical third of the extracted teeth with minimal root curvature (less than 15 degrees) were included. The Nd: YAP laser was used with parameters set to a power of 3W, delivering 300 mJ per pulse. The fiber had a diameter of 200 µm, and the laser operated in pulsed mode at a frequency of 10 Hz, a pulse duration of 150 𝜇s, and an energy density of 955.41 J/cm^2^ per second. The safety of the irradiation parameters was proven in a previous study. The edge of the laser fiber was inserted in the canal until close contact with the fractured file was achieved during all the irradiation procedures. Total elimination and bypass of the instrument were considered a success while partial bypass, non-bypass, or lateral perforation were considered a failure. In addition, the average time required to bypass or eliminate the broken Ni-Ti files was recorded. Scanning electron microscopy (SEM) was used to evaluate physical modifications after irradiation, and X-ray emission spectroscopy was employed to calculate the percentage of nickel and titanium incorporated into the dentinal walls of the canals.

Results

Six samples were excluded from the study. From the 60 included, the success rate was 100%, with 71.66% (n=43) bypass and 28.33% total elimination (n=17) of the fractured instrument. The mean and standard deviation of the measured time for bypassing or completely removing the broken files was 7.463 ± 3.679 seconds. Chemical analysis revealed that Ni and Ti residues were predominantly present in the irradiated canal area with a mass percentage of 5.548 ± 4.621 and 7.371% ± 5.393 for Ni and Ti, respectively, at the impact area and decreased toward the apical region.

Conclusion

This study proposes a promising protocol using Nd: YAP laser for removing fractured Ni-Ti files in teeth with minimal root curvature that is less than 15 degrees.

## Introduction

During root canal treatment and retreatment, different complications can occur while cleaning and shaping canals. Some issues are manageable, but others can greatly impact the treatment outcome and may even result in complete failure in certain cases [1-3]. One of the most common complications affecting the prognosis is instrument fracture within the root canal (RC) during canal shaping [4,5]. All endodontic instruments, including those made from nickel-titanium (Ni-Ti), stainless steel, or carbon steel, are prone to fracture during root canal procedures [1,3]. Several factors contributing to instrument fracture are well-documented in the literature. These include the operator's skill and experience, canal morphology, tooth type, and the cumulative use of the instrument [6-9]. When a Ni-Ti instrument fractures, the prognosis is primarily influenced by factors such as the stage of canal preparation, level of microbial contamination, and location of the fractured instrument [10]. Several techniques and tools have been developed to handle fractured instruments, including ultrasonic devices and hollow tubes with cyanoacrylate adhesive [11], and microtubes combined with needles [12-14]. Surgical approaches involving the removal of the fractured instrument or the entire root segment containing the instrument have also been described [15-19]. It is crucial, however, to weigh the potential benefits of instrument retrieval against the risks, as aggressive dentin removal during these procedures may result in perforation or an increased likelihood of vertical root fracture. Therefore, case-by-case evaluation is essential, especially since no gold standard protocol currently exists for the management of fractured instruments within root canals [20].

Given the Nd: YAP laser's demonstrated ability to cut through metals, it presents a promising solution for addressing fractured instruments in root canals. The laser’s capacity for precise, controlled removal of the broken instrument is advantageous, as it allows for selective ablation while minimizing damage to adjacent healthy tissues. Furthermore, the flexibility of the laser fiber may enable clinicians to limit the removal area to the specific site of interest [21,22]. Investigating the Nd: YAP laser's potential in this context is, therefore, of considerable interest. This in-vitro study aims to assess the effectiveness and safety of a treatment protocol that employs the Nd: YAP laser, with a 1340 nm wavelength, for removing or bypassing fractured instruments located in the apical third of teeth with minimal root curvature (under 15 degrees). Part 2 of this study will evaluate the success rate of the same protocol but on teeth with significant root curvatures (greater than 15 degrees). The null hypothesis posits that the treatment protocol will not be successful in removing or bypassing the fractured instruments.

## Materials and methods

Study design

**Figure 1:**
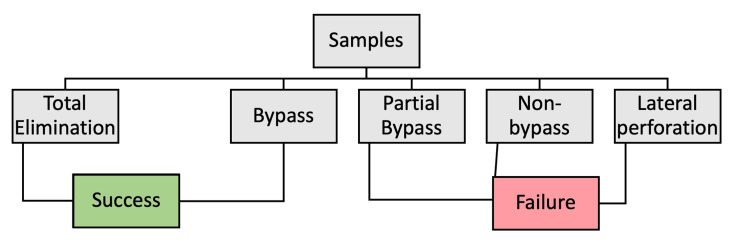
Illustration of the methodology for assessing the results

This study aimed to evaluate the effectiveness of a protocol using an Nd: YAP laser (λ: 1340 nm, LOBEL MEDICAL SAS, Les Roches de Condrieu, France) for removing fractured instruments in root canals of teeth with minimal root curvature (less than 15 degrees). Each experiment's outcome was assessed at the end and classified as either successful or unsuccessful. Success was defined as the complete removal (disintegration) of the fractured files or the successful bypassing of the broken instrument. Unsuccessful outcomes included partial bypass, lateral perforation, or failure to bypass the canal (Figure 1). No ethical approval from the University of Liege was needed, as no patients were involved; all extracted teeth were removed for reasons unrelated to the research, and participants provided written consent allowing future research use of their teeth. Extracted teeth were collected from different clinics. The extraction was made during routine dental care for reasons such as orthodontic treatments and non-restorable teeth. To induce instrument fractures in the root canal (RC), the apical 2 mm of a Reciproc R-25 file (VDW GmbH, München, Germany) was notched with a fine bur to about three-quarters of the file’s thickness, promoting separation in the apical area. The files were inserted using continuous rotation until a fragment lodged and separated, with obstruction verified by a 10 K-file (VDW GmbH). Samples where bypassing was possible were excluded, totaling six exclusions. Working lengths for each canal were calculated, and the laser fiber was positioned in the canal until it made close contact with the fractured file. Radiographs confirmed the fractured instrument's location in the apical third and the fiber’s position in contact with the file. A stopper was placed on the laser fiber at each canal’s working length, and a protocol from a previous study was followed [22]. After each irradiation, a 10 K-file (VDW GmbH) was used to assess the results (complete removal, bypassing, or failure). For all cases, teeth were sectioned for additional visual confirmation. An energy-dispersive X-ray (EDX) spectroscopy analysis (JSM 7500F, JEOL, Tokyo, Japan) was conducted to determine the chemical composition of dentinal canal walls after laser-assisted instrument removal. EDX, a standard method for identifying local chemical composition, measured nickel and titanium levels in the cervical, middle, and apical sections of each canal (impact areas). Six points in each canal section were randomly analyzed, calculating the percentage of atomic mass for Ni and Ti, and statistical analysis was performed to obtain mean and standard deviation values.

Tooth preparation

A total of 66 human permanent teeth (n=66) were used in this study. All teeth were extracted and collected for reasons unrelated to the research. After collection, the specimens were washed, cleaned with a scaler (Satelec, Acteon), and stored in a 0.1% thymol solution at 4°C to inhibit microbial growth. Endodontic preparation was carried out manually using a standardized step-back serial technique with ISO K-Files 10, 15, and 20 (VDW, GmbH) until the working length (WL) of each root was reached. The working length was then measured for each canal.

Inclusion and exclusion criteria

The inclusion criteria consisted of permanent teeth with minimal root curvature (less than 15 degrees), closed apical root formation, and teeth with either a single canal or multirooted teeth exhibiting straightforward canal anatomy as assessed radiographically. The exclusion criteria included the presence of decay in the pulp chamber, radicular decay, previous endodontic treatment, constricted root canals, apices exceeding #25 files, internal resorption, and external resorption.

Procedure to induce instrument fractures

**Figure 2:**
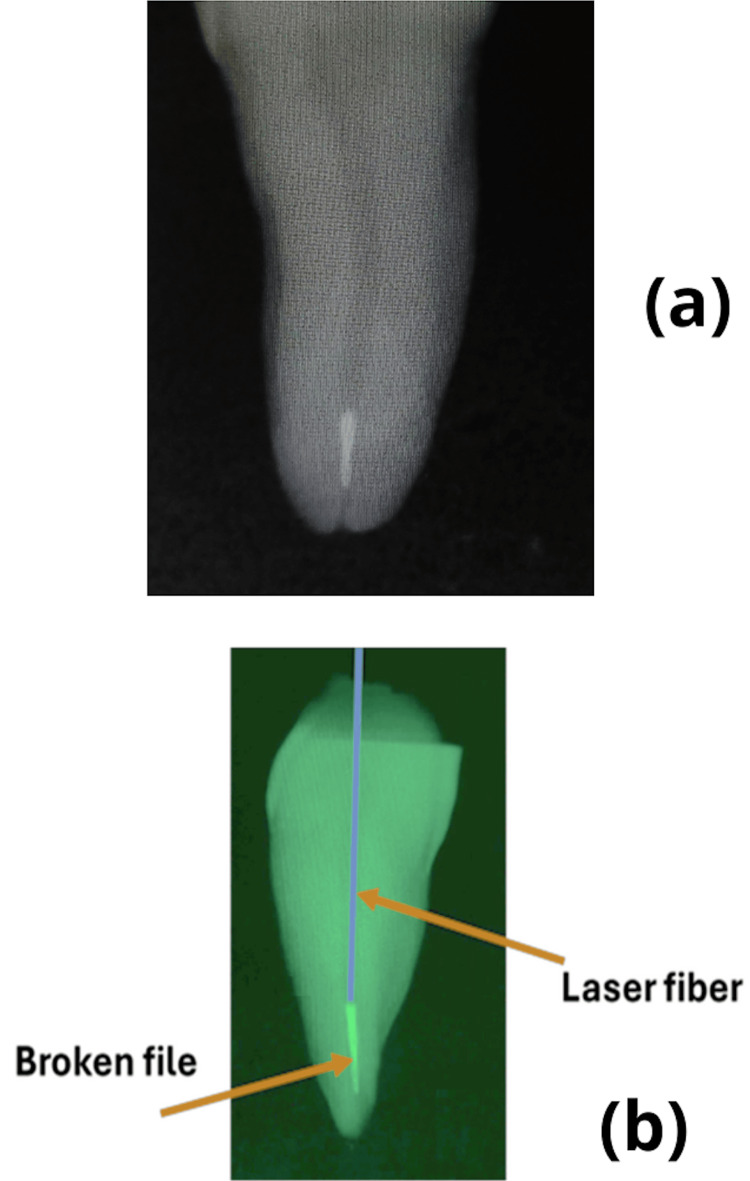
Peri-apical X-rays performed prior to the treatment to confirm the obstruction of the Ni-Ti instrument (A) Presence of a fractured file at the apical third of the root canal. (B) Laser fiber positioned in close contact with a broken file before irradiation.

To create instrument fractures within the RC, a Reciproc R-25 file (VDW GmbH) was notched 2 mm from the tip with a fine bur, cutting to three-quarters of the file’s thickness to encourage separation in the apical region. The files were then inserted using continuous rotary motion until a fragment was lodged and fractured. A 10 K-file (VDW GmbH) confirmed the obstruction. Samples where bypassing was feasible were excluded, leading to six exclusions (n=6). The laser fiber was positioned in the canals until it was in close contact with the fractured file. Parallel radiographs were taken to confirm both the instrument fracture location in the apical third and the fiber’s contact position (Figure 2). A stopper was then placed on the laser fiber at each canal’s working length, following a protocol based on a previous study [23].

Treatment protocol and laser irradiation parameters

Our procedure followed a protocol similar to that used in a previous study [22]. The protocol consists of 3 irradiations of five seconds with 30 seconds of resting time at the end of each irradiation (3x (5s + 30s RT). The procedure was always performed under continuous irrigation by sodium hypochlorite (NaOCl) solution (5.25%). Approximately 6 mL of NaOCl was used for irrigation in each sample. Irradiation parameters were as follows: output power of 3W, 300 mJ per pulse, 955.41 J/cm2, frequency of 10 Hz with 150 µs/pulse, fiber diameter of 200 microns, contact mode. Before irradiation, the laser fiber with a diameter of 200 µm has been inserted in the root canal until having close contact with the broken instrument. During the entire irradiation process, a slight pressure was continuously exerted on the fiber to ensure continuous contact between the fiber and the broken file in the root canal. The laser fiber was equipped with a rubber stopper indicating the working length in each root canal for the operator. The irradiation was conducted until reaching the working length by the fiber or until the end of the 15 seconds of total irradiation time respected for each experiment. Then, a rest of 30 seconds was given.

Evaluation method for protocol success

**Figure 3:**
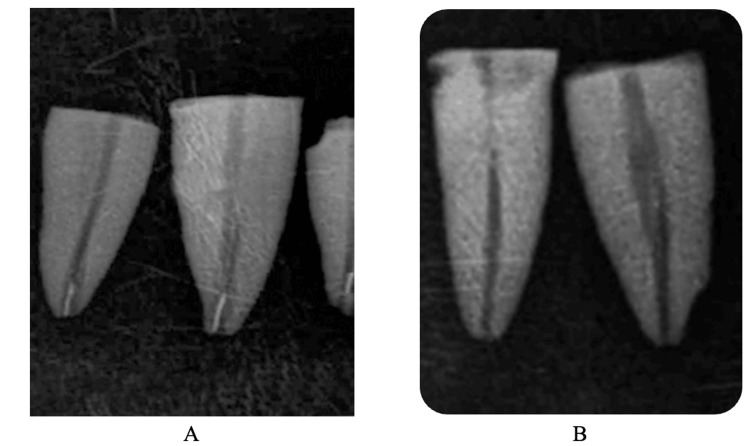
Postoperative X-rays (A) Three samples with a successful bypass of the fractured instrument, showing different remaining dimensions of the Ni-Ti fragment (B): Two samples showing a complete elimination of the broken file.

**Figure 4:**
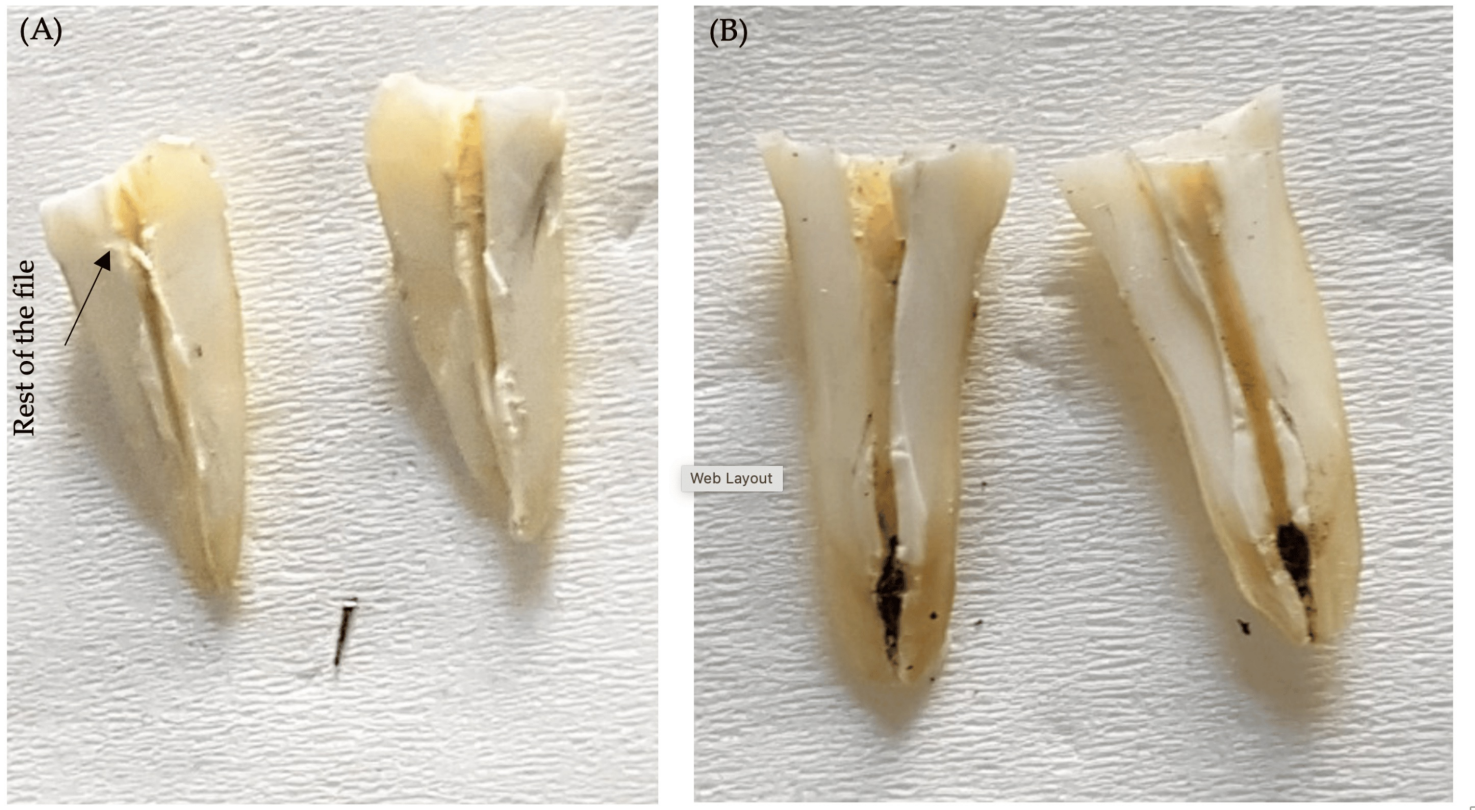
Illustration of the vertical sections performed after treatment to validate the study results (A) Case evaluated as a successful bypass. (B) Case of complete removal of the broken file. Carbonization is observed on the dentinal wall surfaces (black discoloration).

At the end of each irradiation, a 10 K-file (VDW GmbH) was used to evaluate the results (complete removal, bypassing, or failure) (Figure 3). The results were evaluated and classified as either a failure or a success according to the criteria outlined in section 2.1. Then, all teeth were sectioned to allow a second visual confirmation of the results (Figure 4).

Assessment with scanning electron microscopy (SEM)

SEM (JEOL) analysis was conducted to evaluate potential physical changes in the dentinal walls resulting from the irradiation protocol. The specimens were dehydrated in blue silicon with a humidity indicator at room temperature. They were then mounted on aluminum stubs and coated with a 25 nm thick layer of gold using vacuum evaporation in a metallizer (model SCD 005, Bautec, Berlin, Germany). The samples were observed under various magnifications. The thickness of dentinal melting and/or the sealing of root canal tubules after laser irradiation was measured. Five measurements were taken for each specimen, allowing the calculation of the mean and standard deviation of the melted dentinal layer caused by the fractured file irradiation. Statistical analysis was subsequently performed on the SEM results.

Assessment with energy-dispersive X-ray (EDX) spectroscopy

Through EDX (JEOL), the chemical compositions of dentinal canal walls produced by laser-assisted elimination of broken files were analyzed. EDX, which is the standard technique for the local determination of the chemical composition, was used to measure the content of nickel and titanium on dentinal canal walls at the cervical, middle, and apical parts of each canal (impact area). The area of an EDX peak of an element in a sample is directly proportional to the abundance of the elements in the sample: six points were analyzed randomly in each canal part. The percentage in atomic mass (%) of Ni and Ti were calculated and statistical analysis for the mean and std values was carried out.

Statistical analysis

Statistical analyses were achieved using Prism 5® software (GraphPad Software, Inc., San Diego, CA, USA) for the obtained mean and standard deviation of the mean time required for complete removal and/or bypass, the mean and standard deviation of the values of the thickness of the melted dentin measured with the SEM and the mean and standard deviation of the EDX results obtained on the samples. P < 0.05 was considered statistically significant. The confidence level of the study was proposed to be 99% with P < 0.001, which is highly significant. Descriptive statistics, including the means and standard deviations, were calculated. Analysis of variance (ANOVA) tests, coupled with a Newman-Keuls Multiple comparison test (post-hoc test), were used.

## Results

K-file bypass

Six samples were excluded because a bypass was made before the beginning of the treatment (n=6). Of the 60 samples that underwent the treatment protocol, complete elimination of broken files was achieved in 28.33% of cases (n=17) while 71.66% (n=43) showed successful bypass (Table 1). Hence, the success rate of the suggested treatment protocol was 100%. X-rays and root sectioning confirmed the results and were in accordance with the findings. The null hypothesis was rejected.

**Table 1:** Results of the protocol using the Nd Laser for eliminating, bypassing, or failing to bypass the broken instrument Different superscript letters indicate statistically significant differences (A; B; C; D); similar superscript letters indicate no statistically significant difference (A; A).

Group	Success		Failure		
	Total elimination	Bypass	Partial bypass	Lateral perforation	Non-bypass
Minimal root curvature teeth (n = 60)	28.33 % ^A^ (17 teeth)	71.66 % ^B^ (43 teeth)	0	0	0

Average time for bypass or removal

**Figure 5:**
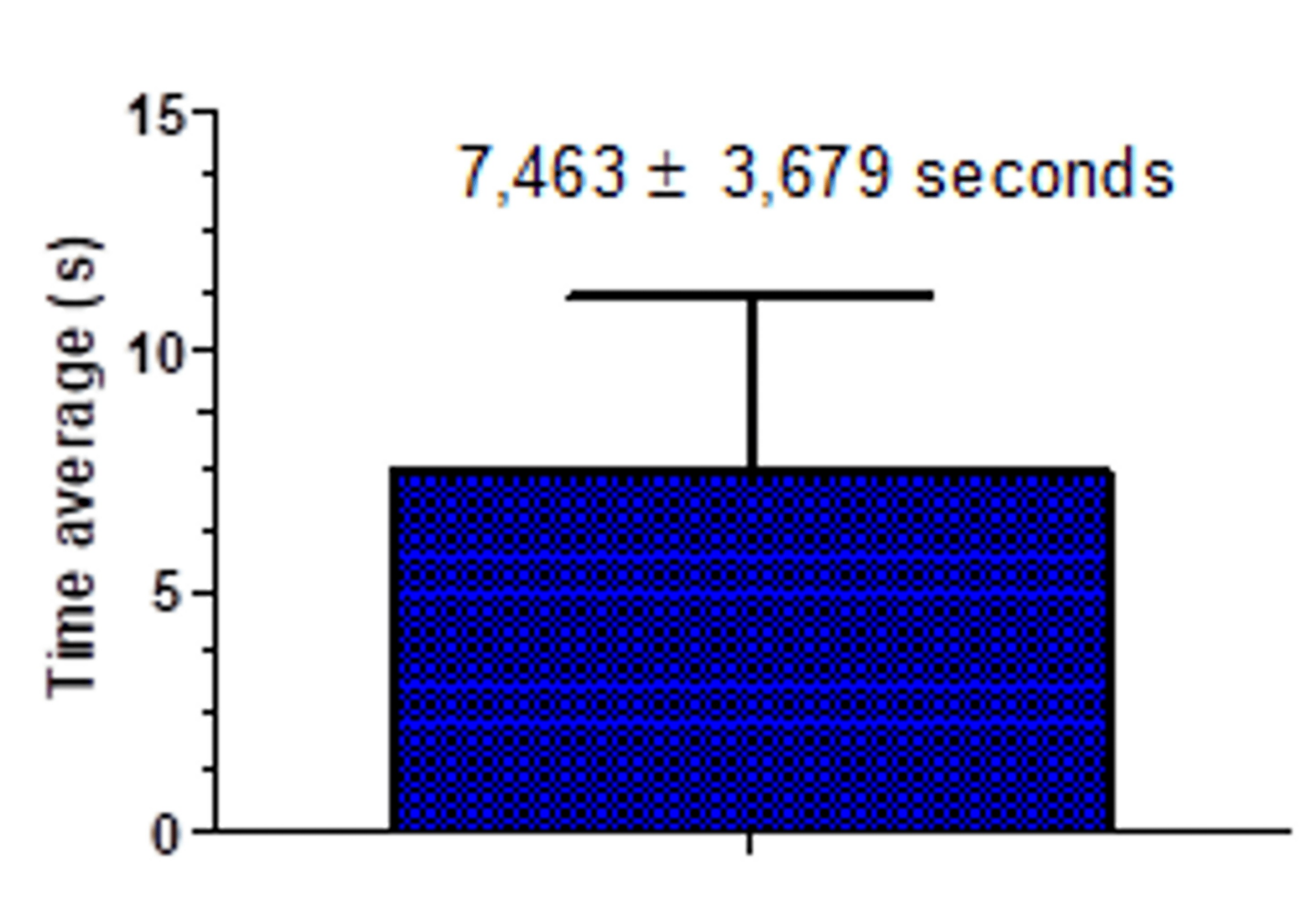
Mean time in seconds required for complete removal and/or bypass

When successful removal or bypass was achieved, the procedure demonstrated a relatively efficient outcome, with the mean total time recorded as 7.463 ± 3.679 seconds. This suggests that, under optimal conditions, the process can be completed in a relatively short duration. The time variation indicates that while the majority of cases were resolved promptly, some instances required slightly more time due to factors such as complexity or technical challenges (Figure 5).

Results of the SEM analysis

**Figure 6:**
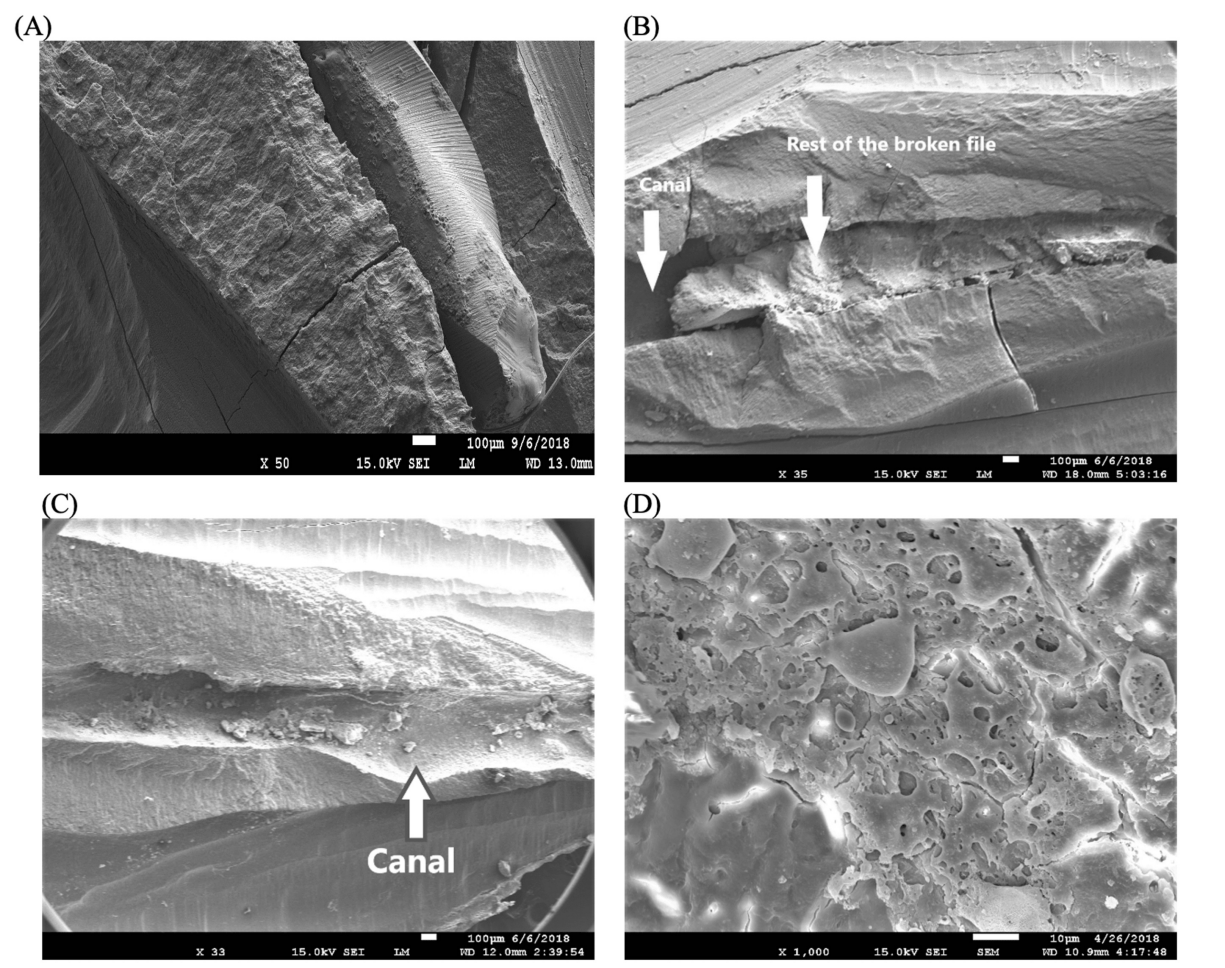
Scanning electron microscopy (SEM) views of different cases (A) SEM image of a root canal showing the broken file before treatment. (B) SEM image of the laser area; the arrows show the bypassed broken file. (C) Case showing a complete removal; the arrow confirms the complete disintegration of the broken file. (D) View of melted dentin in a canal where the file was fully removed.

The SEM views reveal a partial melting of the dentin in the concerned area that was irradiated. Moreover, images of the broken file lodged in the root canal before treatment were seen. Another SEM view illustrated the success of the Nd: YAP laser-assisted treatment in bypassing the fractured file, with minimal disruption to the dentin. In a separate image, the complete removal of the broken file was clearly visible, representing a successful outcome. See Figure 6 for the images of the SEM analysis.

Results of the EDX-ray spectroscopy analysis 

**Figure 7:**
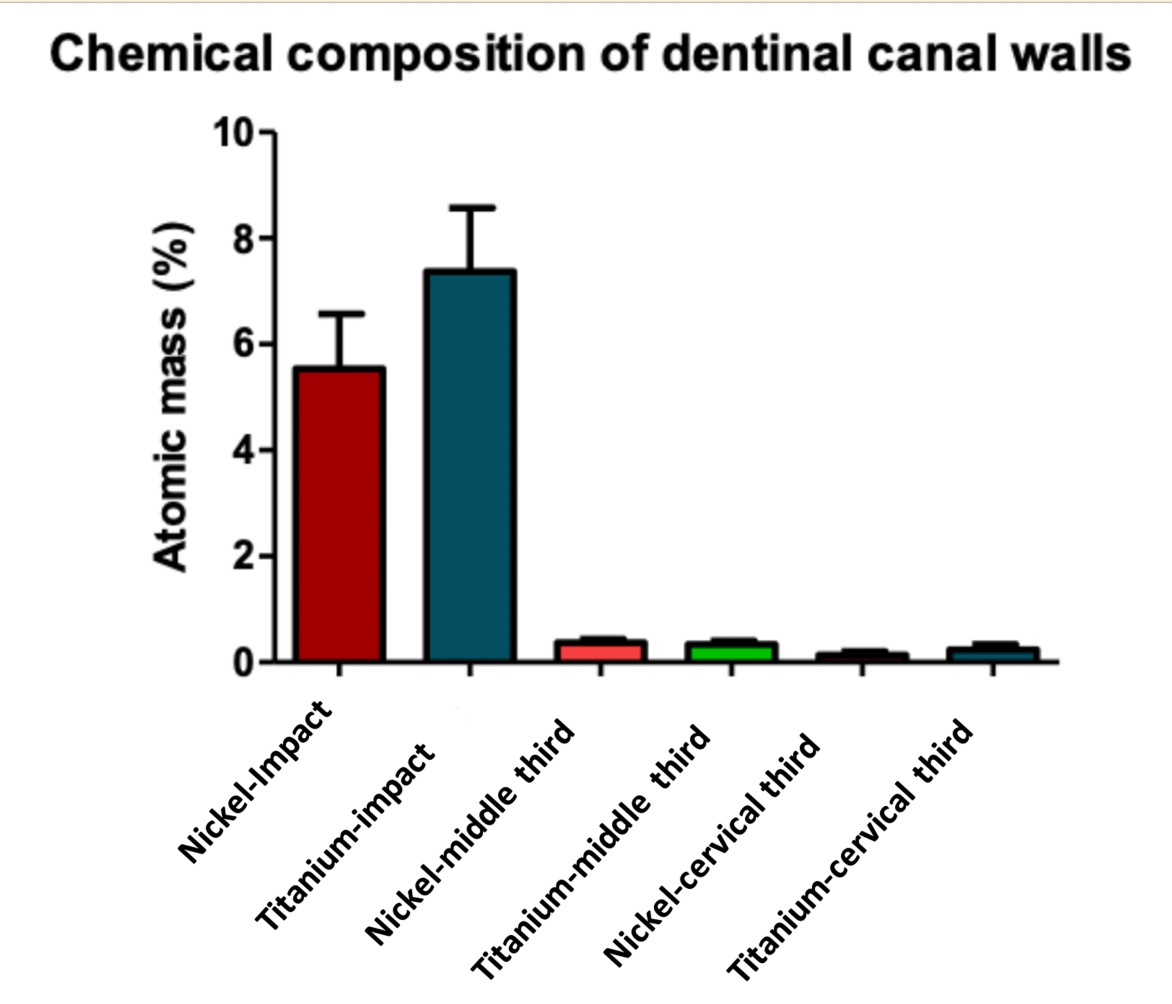
Chemical composition of nickel and titanium in different parts of the dentinal canal walls Values are expressed as atomic mass percentages (%).

The EDX analysis showed the chemical composition of Ni and Ti in dentinal walls with values of 5.548% ± 4.621 and 7.371% ± 5.393 at the irradiation area (impact area) for Ni and Ti, respectively. This presence of Ni and Ti traces decreased above (more coronal) and below (more apical) the impact area. The values of nickel and titanium were, respectively, 0.53% ± 0.23 and 0.31% ± 0.27 coronal to the impact area and 0.13% ± 0.17 and 0.22% ± 0.26 apical to the impact area (Table 2; Figure 7).

**Table 2:** Chemical composition of dentinal walls in nickel and titanium in different parts of the canals Values are expressed as a percentage of atomic mass (%). Different superscript letters indicate statistically significant differences (A; B; C; D and a; b); Similar superscript letters indicate no statistically significant difference (b; b). Std. = standard deviation. Unit = atomic mass (%)

	Impact zone		Coronal to the impact zone		Apical to the impact zone	
	Nickel	Titanium	Nickel	Titanium	Nickel	Titanium
Mean	5.548 ^A^	7.371^ a^	0.3513 ^B^	0.3106 ^b^	0.1318 ^C^	0.2291 ^b^
Std.	4.621	5.393	0.2300	0.2764	0.1786	0.2646

## Discussion

Instrument separation, or the fracturing of endodontic instruments, presents a significant challenge in root canal therapy that requires careful management to minimize its occurrence [3]. When this happens, it can severely affect the cleaning, rinsing, shaping, disinfecting, and filling processes of the root canal, potentially reducing the overall success of the treatment [3-6]. As previously mentioned, there is no universally accepted protocol for managing instrument separation in the root canal. However, many experts agree that the complete removal or bypassing of the fractured instrument is considered a successful approach [24]. Due to the significant impact fractured instruments have on treatment success and the limited number of available methods, ongoing studies aim to identify techniques that can better manage this complication [24].

This ex-vivo study was conducted using a promising approach involving Nd: YAP laser irradiation, which has already shown safe and effective results in previous research [22]. A prior study demonstrated that the best outcomes are achieved when the Nd: YAP laser is used following a protocol that involves three series of five-second irradiations, with a 30-second rest period between each series, using the recommended irradiation parameters [22]. The Nd: YAP laser was chosen primarily due to its interaction with metals. With a wavelength of around 1340 nm, it aligns well with the absorption properties of metals such as nickel-titanium, which lies within the near-infrared range [22].

In this study, EDX analysis revealed the presence of Ni and Ti in the apical, middle, and cervical thirds of the dentinal canal walls, indicating that the laser's interaction with the broken instrument led to its disintegration [22]. This characteristic was beneficial for precisely targeting and manipulating the fractured instrument in the complex root canal system, aiming to achieve controlled interaction while minimizing damage to surrounding tissues. Additionally, the Nd: YAP laser's energy is highly absorbed by the dark color of titanium, enhancing its effectiveness in interacting with the fractured instrument, causing its rapid disintegration and generating a micro-explosion effect that ejects metallic particles from the irradiated area [19,20,25,26]. Moreover, the Nd: YAP laser shows low absorption in dentinal tissue, which is believed to reduce the risk of side effects such as dentin damage or root perforation [19,20].

In this study, the success rate of the protocol was evaluated in minimal root curvature teeth, and the results showed a 100% success rate, demonstrating a high level of effectiveness. The success was in 71.66% of the cases represented by a complete bypass and in 28.33% by a total elimination of the Ni-Ti instrument. Consequently, the protocol is deemed promising for cases involving minimal root curvature teeth. Moreover, in the context of this study, the time required to bypass or eliminate the broken instrument was relatively fast, with a mean value of only 7.463 seconds.

The use of laser technology to enhance endodontic procedures has been documented in the literature. For instance, Bonin et al. utilized a CO2 laser and observed a decrease in dentin permeability following laser exposure in a canine model, suggesting that this can facilitate the shaping process during treatments and retreatments [27]. Paghdiwala et al. illustrated how Er: YAG lasers can alter the mineral components of tubules, melting and fusing them into amorphous particles, effectively eliminating the smear layer typically left by rotary instruments [28]. Tewik et al. suggested that dentin permeability might increase after lasing with KTP/532 [29]. Mashida et al. compared different power settings of KTP and Er: YAG lasers and observed varying effects on the smear layer, which could either disappear or persist depending on the parameters used [30]. Additionally, Moss et al. demonstrated the capabilities of excimer lasers at a 248 nm wavelength on dental hard tissue, resulting in a clean irradiated zone appearance [31]. It can be noted that despite its promising results, there are still relatively few studies in the literature that assess the effectiveness of lasers in the management of broken instruments in root canal treatments. Similar to our study, Farge et al. [32] reported that combining the Nd: YAP laser with conventional instrumentation offers advantages in endodontic retreatments, particularly in removing canal sealers and broken instruments [32]. They demonstrated that the Nd: YAP laser effectively destroys sealer while preserving dentinal walls when in direct contact [32]. Moreover, Farge et al. emphasized the importance of subsequent hand instrumentation to remove carbonized dentin and enlarge the canal wall, preventing the formation of apical plugs due to the accumulation of carbonized dentin or sealer debris [32]. They recommended a one-minute thermal relaxation period between successive phases of irradiation and emphasized continuous contact of the optical fiber with the filling material [32]. Furthermore, their study found that using parameters of 200 mJ and 10 Hz did not induce morphological alterations to the dentin or create crater structures [32].

The safety of this suggested protocol was confirmed in a previous study [23]. Hence, the current study’s results indicated that the protocol is effective in minimal root curvature canals. Part 2 of this study will evaluate the same treatment protocol and assess the success rate, this time on teeth with significant curvatures (greater than 15 degrees).

Among the many advantages of using lasers such as managing oral complications in cancer patients [33], disinfection in periodontics [34], and peri-implantitis [35], their effectiveness can extend to managing broken instruments in endodontics by completely removing or bypassing them. It is important to acknowledge the limitations of this ex-vivo study. For example, the sample size of straight single-rooted teeth was not equal to that of curved teeth, which could be considered a limitation. Additionally, there was no comparison between different types of teeth (incisors, canines, premolars) in terms of effectiveness or success. Such a comparison could have been interesting to understand in clinical scenarios. Conducting the same study in different teeth morphologies, particularly those with root curvatures higher than 10 degrees, is crucial since this presents a more complex anatomical challenge compared to minimal root curvature teeth. Additionally, narrow canals, which significantly increase the risk of instrument separation and complicate retrieval efforts must be studied. Testing our Nd: YAP laser protocol in these challenging scenarios could provide valuable insights into its efficacy and safety in a wider range of clinical situations.

## Conclusions

The suggested treatment protocol for bypassing and/or removing broken instruments can be considered effective, with a very high success rate of 100% in minimal root curvature teeth (less than 15 degrees) represented mainly by a complete bypass of the broken Ni-Ti instrument. It is a safe and relatively fast approach for minimal root curvature teeth. The results of this study should be interpreted with caution, and we invite further studies to investigate the protocol in clinical studies. Additionally, we recommend further studies using the same safe treatment protocol on curved-rooted teeth.
